# Study on Factors Affecting the Performance of a CRISPR/Cas-Assisted New Immunoassay: Detection of Salivary Insulin as an Example

**DOI:** 10.3389/fbioe.2021.752514

**Published:** 2021-11-11

**Authors:** Xiaoting Lin, Gonglei Wang, Long Ma, Guozhen Liu

**Affiliations:** ^1^ Graduate School of Biomedical Engineering, University of New South Wales, Sydney, NSW, Australia; ^2^ School of Life and Health Sciences, The Chinese University of Hong Kong, Shenzhen, China; ^3^ State Key Laboratory of Food Nutrition and Safety, Key Laboratory of Industrial Microbiology, Tianjin Key Laboratory of Industry Microbiology, Ministry of Education, National and Local United Engineering Lab of Metabolic Control Fermentation Technology, China International Science and Technology Cooperation Base of Food Nutrition/Safety and Medicinal Chemistry, College of Biotechnology, Tianjin University of Science and Technology, Tianjin, China

**Keywords:** biosensing, CRISPR/Cas, ELISA, sensitivity, insulin detection

## Abstract

The clustered regularly interspaced short palindromic repeat (CRISPR)/Cas is now playing a significant role in biosensing applications, especially when the trans-cleavage activity of several Cas effectors is discovered. Taking advantages of both CRISPR/Cas and the enzyme-linked immunosorbent assay (ELISA) in analytical and clinical investigations, CRISPR/Cas-powered ELISA has been successfully designed to detect a spectrum of analytes beyond nucleic acid. Herein, we developed a CRISPR/Cas12a-assisted new immunoassay (CANi) for detection of salivary insulin as an example. Specifically, factors (antibody selection, temperature, and assay time) affecting the CRISPR/Cas-based ELISA system’s performance were investigated. It was observed that the concentration of blocking solution, selection of the capture antibody pairs, and the sequences of triggering ssDNA and guiding RNA affected this immunoassay sensitivity. In contrast, the preincubation of CRISPR/Cas12a working solution and pre-mixture of detection antibody with anti-IgG–ssDNA did not show influence on the performance of CANi for the detection of insulin. Under optimized conditions, the sensitivity for detection of salivary insulin was 10 fg/ml with a linear range from 10 fg/ml to 1 ng/ml.

## Introduction

Molecular diagnostics have played essential roles in life sciences, biosecurity, food safety, and environmental monitoring (Choi et al., 2016; Mumford et al., 2016). With the continuous threat of the COVID-19 pandemic to global public health, supersensitive bioassays are becoming critical in molecular diagnostics. Immunoassays are the most popular assays for molecular diagnostics (Cox et al., 2019; Maggio, 2018; Poschenrieder et al., 2019; Organization (W.H, 2020). Various state-of-the-art technologies were combined with the traditional immunoassay and applied in clinical diagnostics, such as a single-molecule array (Simoa) (Mathian et al., 2019; Cohen et al., 2020) and rolling circle amplification (RCA) (Bhat and Rao, 2020; Hadi et al., 2020). Among them, enzyme-linked immunosorbent assay (ELISA) is the gold standard of *in vitro* diagnostics to analyze biomarkers and important analytes in healthcare and diversified analytical settings (Tabatabaei et al., 2020). Compared to other immunoassay methods, ELISA has many advantages, such as being sensitive, specific, and high throughput (Tighe et al., 2015). However, because of the limited catalytic efficiency of horseradish peroxidase (Acharya et al., 2013), traditional ELISA is not sensitive enough to analyze low-abundant analytes such as hormone (insulin), cancer biomarkers, cytokines, and chemokines, which are in the picogram range in clinical samples at the early stage of the disease. For example, it is well-known that the normal fasting insulin levels in the serum range between 0.17 and 1.34 ng/ml (Carmina et al., 2019). Significantly, the lowest concentrations of salivary insulin and serum insulin in children are only approximately 0.0048 and 0.072 ng/ml, respectively (Fabre et al., 2012). Thus, a supersensitive assay is in high demand.

To further increase the sensitivity of ELISA, CRISPR/Cas-based sensing systems were successfully used to integrate with ELISA for the detection of a spectrum of analytes beyond nucleic acids with supersensitivity and specificity (Li et al., 2019a; Dai et al., 2020; Peng et al., 2020; Zhou et al., 2020). Utilizing collateral cleavage of the non-specific ssDNA reporter (termed *trans*-acting cleavage), initiated by the recognition and cleavage of the target DNA by CRISPR RNA (crRNA), CRISPR type II, V, and VI RNA-guided nucleases (Csm6, Cas12a, and Cas13) have been used for the detection of a range of analytes through fluorescent transduction systems (Chen et al., 2018; Gootenberg et al., 2018). Various advanced CRISPR/Cas-based biosensing systems have acquired improved multiplex detection performance, sample treatment, and solid surface compatibility (SHERLOCKv2, HOLMESv2, and HUDSON) (Gootenberg et al., 2018; Myhrvold et al., 2018). For example, HOLMES (Li et al., 2018) detection systems used crRNA-guided Cas12a enzymes for ultrasensitive and rapid detection of target DNA and SHERLOCK (Gootenberg et al., 2018) for target RNA. The LOD (limit of detection) level of both detection systems has reached femtomolar levels (Gootenberg et al., 2018; Li et al., 2019a; Li et al., 2019b; Liang et al., 2019; Bonini et al., 2021). Recently, a CRISPR/Cas-based biosensing system has been integrated with ELISA to detect nucleic acid analytes and protein targets (Chen et al., 2020; Li et al., 2021). Chen et al. (2020) utilized the CRISPR/Cas13a system combined with RNA polymerase and antibody to enhance the signal, which increased the detection sensitivity of proteins to the fM level. However, the RNA reporter used in this assay may be easily degraded during the detection process, interfering with the detection results. The nicking endonuclease coupled with the CRISPR/Cas13a system could also realize dual signal amplification for protein detection. Li et al. (2021) used the CRISPR/Cas12a system for biosensing to detect proteins, small molecules, and tumor cells at the sub-attomolar level by the proposed method through a series of rational design of activator DNA. Liu *et al.* brought together the advantages of CRISPR/Cas biosensing with broad applicability of sandwich ELISA to develop a supersensitive immunoassay to increase the LOD level of the analytes to femtogram (Liu et al., 2021). However, this system was not stable. Although these CRISPR/Cas-powered ELISA has been successfully used to detect a range of analytes with some detection systems reaching a high sensitivity level, no report specifically studied the factors affecting this sensing system providing guidance for future assay development.

In this study, we designed a CRISPR/Cas12a-assisted new immunoassay (CANi). Specifically, we focus on studying the effects of parameters on the performance of CANi using insulin as an analyte model. It was observed that the concentration of blocking solution, selection of the capture antibody pairs, and the sequences of triggering ssDNA and guiding RNA affected this immunoassay sensitivity. However, other experimental conditions, such as the preincubation of CRISPR/Cas12a working solution and pre-mixture of detection antibody with anti-IgG–ssDNA, did not influence the performance of CANi for detection of insulin. This study will provide tutorial guidance for designing the CRISPR/Cas-based immunoassay for sensitive detection.

## Results and Discussion

### Principle and Establishment of CANi

**FIGURE 1:**
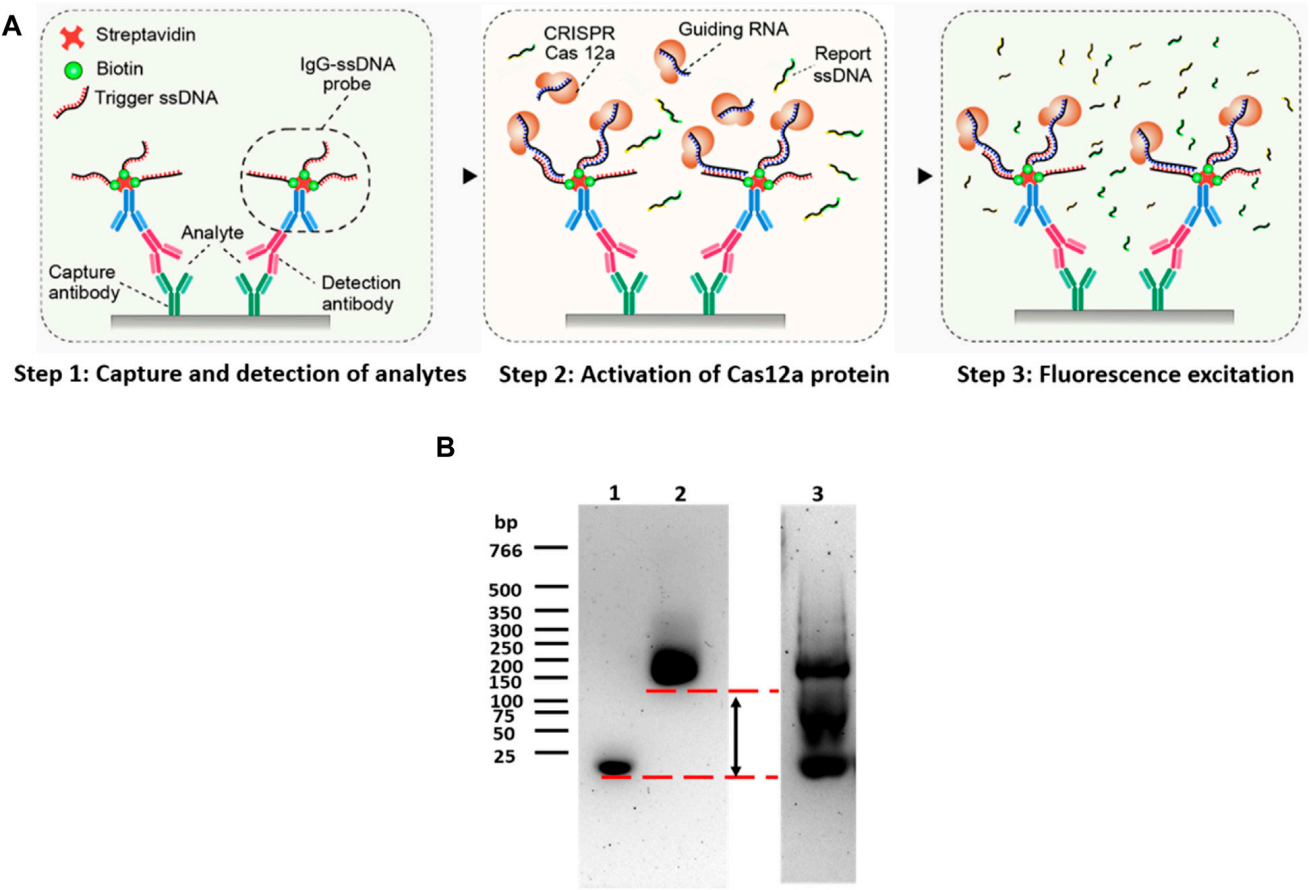
Principle of CANi. **(A)** Principle of the CRISPR/Cas12a-assisted new immunoassay (CANi). **(B)** Electrophoretic mobility shift assay (EMSA). Lane 1 was triggering ssDNA, lane 2 was the IgG–ssDNA probe, and lane 3 was IgG–ssDNA probe before purification.

CANi was designed according to conventional sandwich immunoassay (Figure 1A), which measured the antigen between two layers of antibodies (capture and detection antibody) (Itoh et al., 2002; Tabatabaei et al., 2020). The capture antibody was immobilized on a 96-well polystyrene surface to capture the analytes of interest. The details of the antibody used in the experiments were reported in the Supplementary Table S3. The detection antibody was used to bind the analytes and then was recognized by the anti-IgG–ssDNA probe. The target analytes must contain at least two antigenic sites capable of binding to antibodies. Antibodies are critical to an ELISA and provide the basis for the assay’s specificity and sensitivity (Itoh et al., 2002). Polyclonal antibodies were used as the capture antibody in the assay to capture as much of the antigen in the sample as possible. The other reason was to avoid false positives. The probe used in the assay was fabricated with anti-IgG, which can recognize all IgG from the same host. Thus, the free capture monoclonal antibodies from the same host immobilized on the detection surface might be recognized by the probe and then activate the CRISPR/Cas12a system.

The triggering ssDNA was conjugated in the anti-IgG-ssDNA probe, and the sequences of triggering ssDNA were reported in the Supplementary Table S1. After adding the CRISPR/Cas12a reaction mixture, the CRISPR/Cas12a targeted the triggering ssDNA by its complementary guiding RNA. The Cas12a protein was then activated and collaterally cleaved the quenched fluorescent reporters linked by ssDNA, releasing the highly amplified fluorescence signal related to the target analyte amount (Gootenberg et al., 2017; Gootenberg et al., 2018; Li et al., 2018). Agarose gel electrophoresis was used to assess the purity of the anti-IgG–ssDNA probe (Figure 1B, lanes 2 and 3). Before purification (Figure 1B, lane 3), a strong band was located on 20 bp, indicating that unattached triggering ssDNA was mixed with the anti-IgG–ssDNA probe. The strong 20-bp band was disappeared suggesting that the anti-IgG–ssDNA probe was purified after centrifugation-filtered using a low-binding PES filter (Figure 1B, lane 2). EMSA assessed the efficiency of the anti-IgG–ssDNA probe using agarose gel (Figure 1B). Triggering ssDNA 1 was conjugated with an anti-mouse IgG antibody. The significant shift indicated that ssDNA had been conjugated with IgG. The binding of ssDNA to the antibody caused the significantly delayed movement on agarose gel compared to the pure ssDNA oligos (Figure 1B, lanes 1 and 2).

The IgG–ssDNA probe can bind to the detection antibody and CRISPR/Cas12a protein, which can be regarded as the bridge between the analytes and CRISPR/Cas12a protein. The success of the construction of the IgG–ssDNA probe would determine the success and the sensitivity of this immunoassay (CANi). Therefore, it was necessary to confirm the conjugation of ssDNA and IgG. The DNA electrophoretic mobility shift assay (EMSA) had been widely used to study proteins binding to the DNA oligonucleotide (Holden and Tacon, 2011). The technique was based on the observation that protein–DNA complexes migrate more slowly than free DNA molecules when subjected to non-denaturing polyacrylamide or agarose gel electrophoresis (Kerr, 1995). Gel electrophoresis separated DNA fragments not by charge but by size in a solid support medium, such as an agarose gel (Yılmaz et al., 2012). The negatively charged DNA would migrate toward the bottom end with the positive charge. The migration rate was proportional to size: smaller fragments move faster and wind up at the bottom of the gel (Magdeldin, 2012). Therefore, DNA oligonucleotides that were conjugated with protein would migrate a short distance when compared to the same bare DNA oligonucleotides.

**FIGURE 2:**
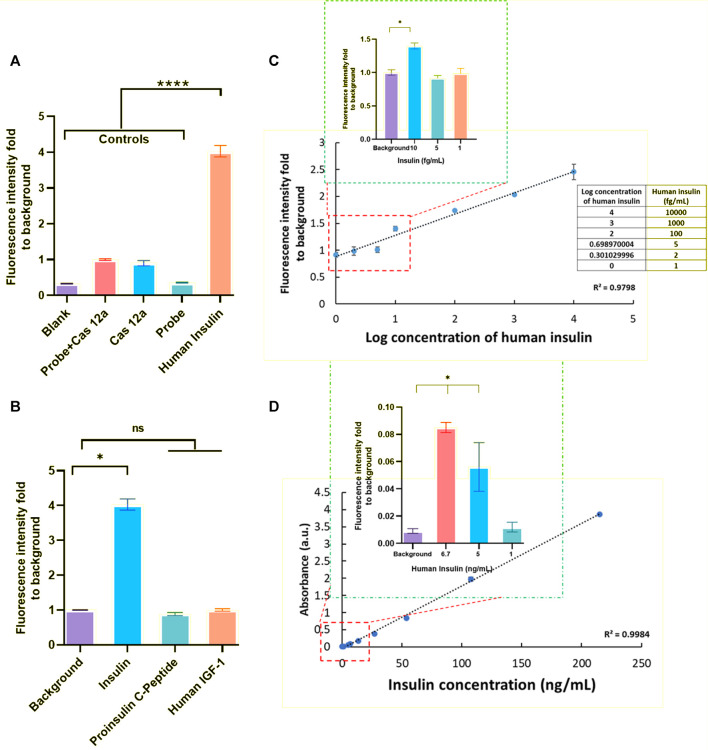
Principle establishment of CANi. **(A)** Detection of human insulin (1 ng/ml) by CRISPR/Cas12a-assisted new immunoassay (CANi). Adding the probe and/or Cas12a reaction mix to the assay system was used as the control group for this assay. The triggering ssDNA 1 and guide RNA 1 and antibody pair #1 (Supplementary Tables S1, S3) were applied in this assay. **(B)** Specificity test for human insulin. **(C)** Calibration curve of human insulin (ranging from 1 ng/ml to 1 fg/ml), and the limits of detection (LOD) for human insulin was 10 fg/ml. **(D)** Verification test for human insulin by ELISA. The LOD for detection of human insulin was 5 pg/ml by ELISA. * indicated significant differences (*p* < 0.05) and **** indicated significant differences (*p* < 0.001) analyzed by two-way ANOVA.

In order to assess the ability and sensitivity for detecting the analytes of the CRISPR/Cas12a-assisted new immunoassay (CANi), recombinant human insulin (I2643, Sigma Aldrich) was selected as a model analyte for this assay (Figure 2A). Anti-human insulin polyclonal antibody (ab53591, Abcam) and anti-human insulin monoclonal antibody (ab6995, Abcam) were applied in the assay as capture antibody and detection antibodies, respectively. The triggering ssDNA 1 and guiding RNA were used in this assay (Supplementary Table S1 **)**. The sequences and concentrations of the anti-IgG–ssDNA probe and guiding RNA used in the assay had been optimized and is reported in the Supplementary Figure S1. The fluorescence signal was nearly four times higher than the control groups, suggesting that human insulin was detectable by this assay. The system specificity test was studied by using human proinsulin C-peptide and human IGF-1. Compared to the presence of other interfering analytes, the significantly higher fluorescent signal intensity for detecting the positive human insulin sample demonstrated the specific response of CANi to its target analytes only (Figure 2B).

With each increase in human insulin concentration, a significantly higher fluorescent signal intensity was observed between different concentrations, which indicated the potential for quantitative analyte detection (Figure 2C). This CANi system can reach 10 fg/ml in detecting human insulin. However, the detection limit of insulin for the commercialized human insulin ELISA kit was observed to be 5 ng/ml (Figure 2D). Therefore, this CRISPR/Cas12a-powered ELISA can significantly increase the sensitivity by 3 orders (Figure 2).

It had been reported that the LOD of IFN-γ in Sioma reached 0.69 fg/ml (Llibre et al., 2018). However, the Sioma assay needs to conduct a highly complex protocol to obtain the high-affinity autoantibodies and activate the magnet beads, which was not user-friendly. An AuNP-RCA biosensor had been used for pathogen detection (Shi et al., 2014), which could identify 0.5 pM of synthetic oligonucleotides or 0.5 ng/ml of genomic DNA. Even so, RCA only could detect the circular molecules of DNA or double-stranded DNA (Hadi et al., 2020), which limited its application. Factors affecting the assay performance is discussed in the following section:

### Streptavidin–Biotin Binding

**FIGURE 3:**
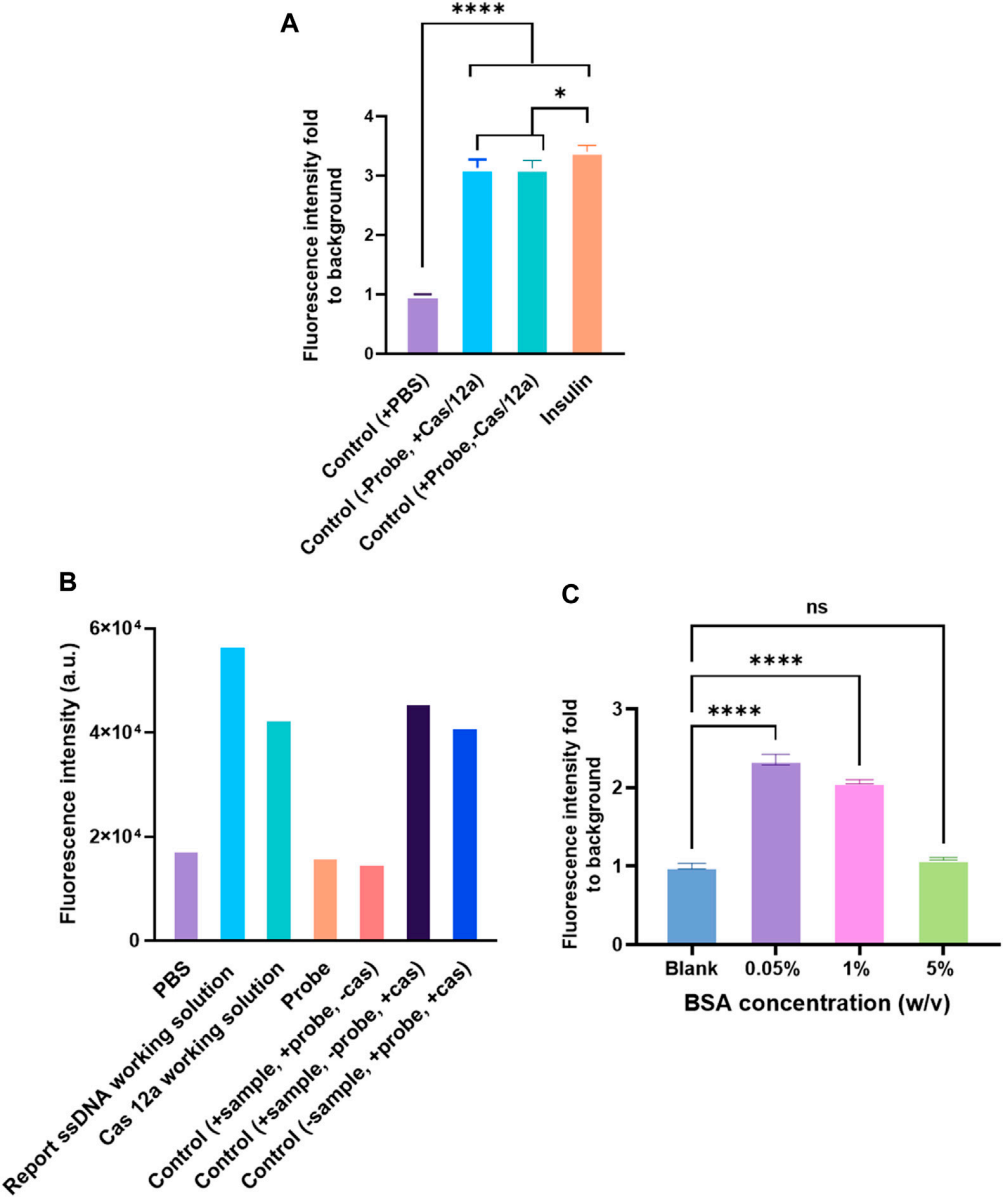
Factors affecting the assay performance. **(A)** Detection of human insulin (1 ng/ml) by CRISPR/Cas12a-assisted new immunoassay (CANi). The polystyrene surface was coated with 10 μg/ml streptavidin first, and then the biotinylated capture antibody (ab53591, Abcam) was immobilized on the surface by binding to streptavidin. **(B)** Background signal analysis. The probe was fabricated with a biotinylated polyclonal antibody. **(C)** Blocking solution optimization. Selection of capture and detection antibody pair.

First, the influence of streptavidin coated on the polystyrene surface was investigated. Streptavidin (10 μg/ml) was coated on the surface before immobilizing the capture antibody. Next, a biotinylated anti-human insulin polyclonal antibody (ab53591, Abcam) was used as a capture antibody. The binding of biotin to streptavidin is one of the most robust non-covalent interactions known in nature. However, coating streptavidin and using biotinylated polyclonal antibodies as the capture antibody had been proved to lead to a significantly higher background (Figure 3A). The reason could be because of the method used to construct the anti-IgG–ssDNA probe (Figure 3B). The ssDNA that fabricated onto the probe had biotin labeled on its 3′ and thus was conjugated with anti-IgG *via* streptavidin. As a result, streptavidin in the probe mixture would bind to the biotinylated polyclonal antibody directly, which had been immobilized on the detection surface, even no analyte is captured by the polyclonal antibody. Therefore, it suggested using the capture antibodies in the assay without the biotin labeling.

### Blocking Solution Concentration

The blocking step in ELISA was vital because it prevented the binding by blocking the leftover spaces over the solid surface after immobilizing a capture biomolecule (Ahirwar et al., 2015) and minimized false-positive results (Maggio, 2018). Numerous chemical and biological reagents, such as Tween 20, polyethylene glycol, casein, milk proteins, serum albumins, and fish serum, have been used for blocking purposes (Reimhult et al., 2008). Among the available blocking reagents, BSA was the most preferred and commonly used blocking reagent because of its low cost and reduced steric hindrance of specifically binding proteins (Reimhult et al., 2008; Jeyachandran et al., 2010a). The BSA layer exhibited a 90–100% blocking efficiency on a hydrophobic and 68–100% on a hydrophilic surface (Jeyachandran et al., 2010b). However, to achieve the blocking sufficiency, the concentration of the BSA solution had to be optimized. The 0.05% BSA led to a high background signal (Figure 3C) in CANi. With the increasing concentration of BSA, the background signal decreased; 5% BSA was proven to reduce the background signal to the acceptable level (Figure 3C). Therefore, 5% BSA was used for further experiments.

**FIGURE 4:**
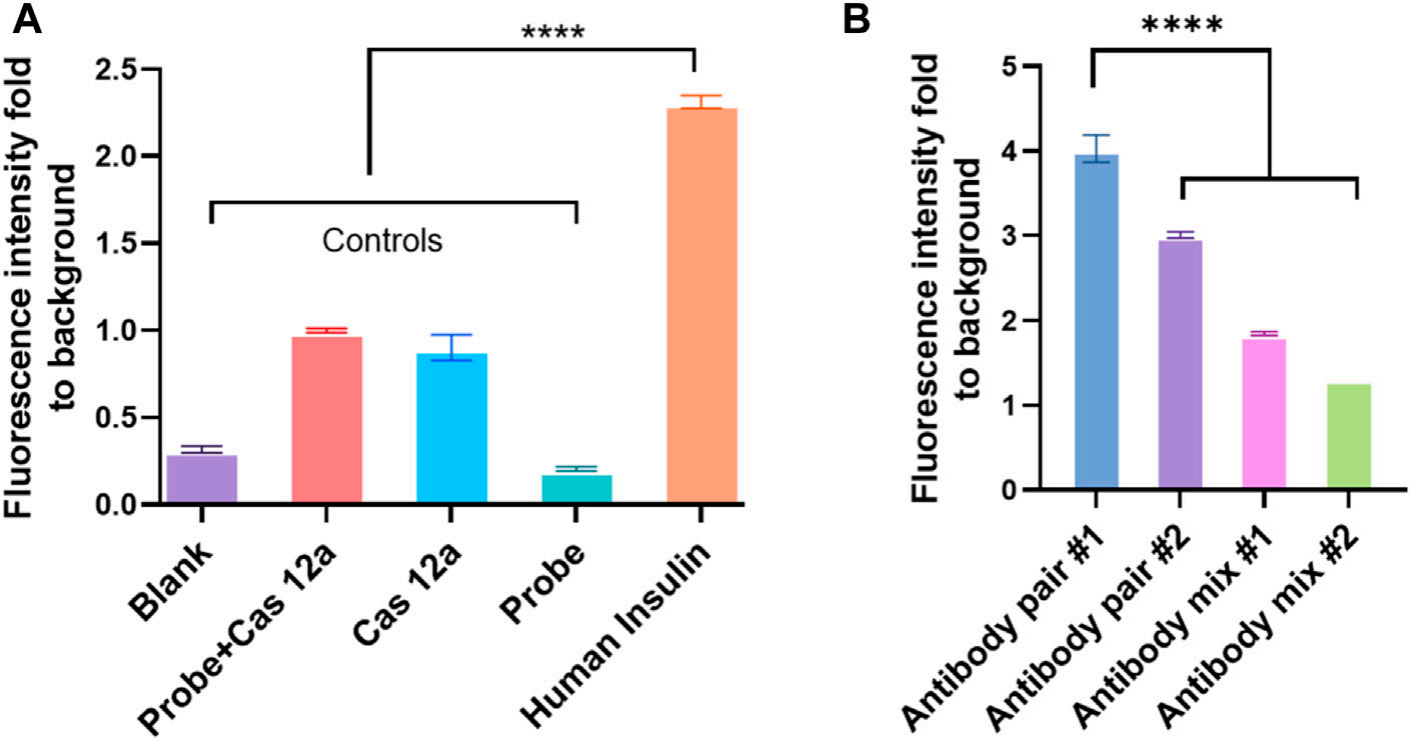
Different antibody pairs affect the efficiency of CANi. **(A)** Detection of human insulin by using antibody pair #2 (Supplementary Table S3). **(B)** Different antibody pairs influent the detection of the analytes by CANi. The capture antibody was from pair # 1, and the detection antibody was from pair # 2 in antibody mix 1. The capture antibody was from pair # 2, and the detection antibody was from pair # 1 in the antibody mix 2. **** indicates significant differences (*p* < 0.0001) analyzed by two-way ANOVA. Design of anti-IgG–ssDNA probe and guiding RNA.

This study also assessed two pairs of capture and detection antibodies for human insulin. The details of the antibody pairs had been listed in the Supplementary Table S3. The first pair of antibodies was approximately four times the fluorescence signal than the control (Figure 2). Meanwhile, the other pair of antibodies produced three times the fluorescence signal level as the control group (Figure 4A). One of the reasons why the signal readout was different when using the other antibody pairs to detect the same analyte could be the different capture antibodies used in the system. The capture antibodies in both antibody pairs were polyclonal. In pair #1, capture antibody (ab53591, Abcam) could detect the recombinant human insulin (Wei et al., 2018); in contrast, the immunogen of the anti-human insulin polyclonal antibody in pair #2 (NBP1-87485, Novus Biologicals) was part of the full length recombinant human insulin (Ghazizadeh et al., 2017). Therefore, there might be a difference in binding ability between these two polyclonal antibodies when human insulin was exposed to the system (Figure 2B).

Additionally, a monoclonal antibody in pair #1 (ab6995, Abcam) and a monoclonal antibody in pair #2 (NBP100-73008, Novus Biologicals) were used as the detection antibodies. Unlike the polyclonal antibody, a monoclonal antibody can have a monovalent affinity, binding only to the same epitope (Davies and Chacko, 1993). The antibody in pair #1 (ab6995, Abcam) was mouse monoclonal (clone K36aC10) against human insulin, and the antibody in pair #2 (NBP1-87485, Novus Biologicals) was mouse monoclonal (clone 3A6) against human insulin. The most common monoclonal class was IgG, which had two chains: the light and heavy chains (Davies and Chacko, 1993; Wang et al., 2007). The variable domains (V_L_ and V_H_) are located at the N-terminal of the heavy and light chains (Wang et al., 2007). The binding site or antibody-combining site for analytes was constructed when the V_H_ and V_L_ domains were paired and formed the hypervariable loops (Janewa et al., 2001). Three hypervariable loops determined antigen specificity, and the combination of the heavy and the light chains determined the final antigen specificity (Janewa et al., 2001). Therefore, the binding sites could be affected by the structure of IgG; therefore, the different binding sites might influence the binding ability of monoclonal antibodies (Figure 2B).

The fluorescence intensity decreased dramatically when two pairs of antibodies were mixed, and the mixture was used as the detection antibody (Figure 4B). It was well-known that the paired antibodies need to be matched in the sandwich ELISA (Tabatabaei et al., 2020). This ensures that the antibodies detect different epitopes on the target protein and do not interfere with the other antibody binding (Tabatabaei et al., 2020). Antibody affinity and avidity can be considered the main factors for the binding efficiency between the analyte and antibody (Aikawa and Ducheyne, 2011). Antibody affinity is the strength of the bond between the analyte and antibody. It was determined by the closeness of the stereochemical fit between antibody sites and analyte determinants. It also depends on the contact area and the distribution of charged and hydrophobic groups (Aikawa and Ducheyne, 2011). The different affinity of these two monoclonal antibodies may be one reason why the fluorescence intensity varied. Monoclonal antibodies have a monovalent affinity. Unlike the polyclonal antibody, which can bind to multiple epitopes, the monoclonal antibody only binds to the same epitope. If the epitopes were covered by the capture antibody (polyclonal antibody) in CANi, the monoclonal detection antibody would not bind to the analytes. Therefore, the signal dropped dramatically (Figure 2B).

**FIGURE 5:**
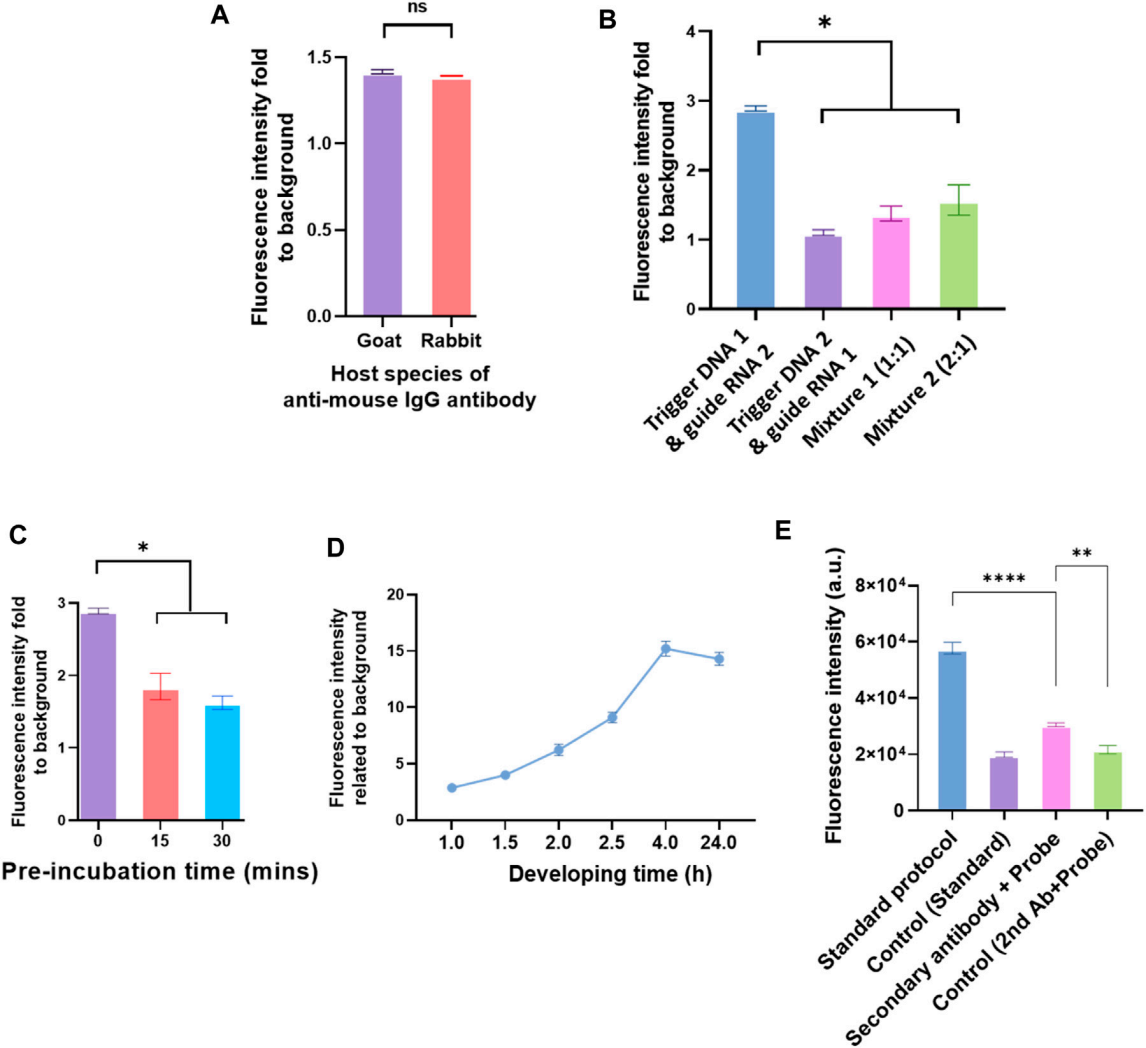
Different elements in the IgG–ssDNA probe conjugation and elements in the Cas12a reaction mix affect the detection of analytes by CRISPR/Cas12a-assisted new immunoassay (CANi). **(A)** Detection efficiency of probe conjugated with different host species of anti-mouse IgG antibodies. **(B)** Influence of triggering ssDNA and guiding RNA sequences on detecting the analytes by CANi. The sequences of triggering ssDNA and guiding RNA were listed in the supplementary information Supplementary Table S1. Mixture 1 was mixed triggering ssDNAs 1 and 2 with ratio 1:1, and mixture 2 was mixed triggering ssDNAs 1 and 2 with ratio 2:1. **(C)** Preincubation decreased the efficiency of detecting the analytes. **(D)** Cas12a reaction time, ranging from 1 to 24 h. **(E)** Detection efficiency of adding the detection antibody and probe separately or adding the complex of detection antibody and probe. * indicates significant differences (*p* < 0.05), ** indicates significant differences (*p* < 0.005), and **** indicates significant differences (*p* < 0.0001) analyzed by two-way ANOVA.

The anti-IgG–ssDNA probe had two main functions in the system. The first one was to recognize the detection antibody that binds to the analytes. The other one was identified by guiding RNA to guide Cas12a protein *via* the probe’s complementary triggering ssDNA. It was observed that IgG from the different hosts did not affect the immunoassay effect (Figure 5A). The sequences of triggering ssDNA and guiding RNA used in the assay are listed in the Supplementary Table S1. The sequence of triggering ssDNA was designed according to the guiding RNA sequence, which was vital to this immunoassay. A quenched fluorescent ssDNA reporter was used in the reaction system. The sequence is listed in the Supplementary Table S1 **.** The Cas12a/guiding RNA binary complex forms a ternary complex with the target DNA, which would then *trans*-cleave the non-targeted ssDNA reporter in the system (Mohr et al., 2016).

Two different guiding RNAs (also two different complementary triggering ssDNA) were applied in the CANi (Supplementary Table S1). The fluorescence signal level produced by the guiding RNA 1 group was significantly higher than the guiding RNA 2 group (Figure 5B). It was also found that by adding the triggering ssDNA 2 with guiding RNA 2 or the mixture of triggering ssDNAs 1 and 2 with the guiding RNAs 1 and 2, the fluorescence intensity decreased approximately one-third and a half. Different fluorescence intensities indicated that the changed sequence of the triggering ssDNA affected the binding between ssDNA and guiding RNA and further influenced the capability to detect the analytes. Therefore, designing an appropriate guiding RNA was an essential element of the CRISPR/Cas12a biosensing system. The most commonly used guiding RNA was approximately 100 base pairs in length by altering the 20 base pairs toward the 5′ end. A reduced length of guiding RNA presented higher cleavage specificity (Li et al., 2018). An 18-nt guiding sequence resulted in more than a 2-fold difference in fluorescence signals than a 24-nt guiding RNA. However, when 15-nt guiding RNA was used, the signals decreased (Li et al., 2018). The sequences of guiding RNA used in the CANi were both 42 nt in length (Supplementary Table S3). Therefore, the length of guiding RNA might not affect this biosensing system. However, the secondary structure can influence guiding RNA effectiveness, and some structural elements had been shown to have a beneficial influence on guiding RNA effectivity (Doench et al., 2014; Wong et al., 2015; Bruegmann et al., 2019). It had been reported that a hairpin structure onto the spacer region enhanced the specificity of the CRISPR system (Kocak et al., 2019). The sequences of guiding RNA used in the CANi had different secondary structures, which are reported in Supplementary Figures S2, S3. Guiding RNA 1, which complemented with triggering DNA 1, had a hairpin structure. In contrast, guiding RNA 2 did not contain any secondary structure. Our result showed that the guiding RNA’s hairpin structure highly improved the signal level (Figure 5B). In the meantime, with an increasing amount of the guiding RNA 1 in the mixture, the fluorescence intensity enhanced, which was consistent with that in the literature (Doench et al., 2014; Wong et al., 2015; Bruegmann et al., 2019).

### Preincubation of CRISPR/Cas12a Reaction Mix

It was reported that preincubation of Cas13a in the CRISPR/Cas system helped the enhancement of sensitivity (Fozouni et al., 2021). However, our study showed that preincubating did not improve the CANi efficiency. The fresh working solution of Cas12a had the highest efficacy. By extending the preincubating time, the fluorescence intensity decreased (Figure 5C). Similarly, a longer incubating time than 4 h after adding Cas12a did not improve the signals (Figure 5D). The fluorescence signal kept increasing after adding the Cas12a reaction mix until 4 h. After that, the signal level flattened until 24 h. It had been well-known that Cas12a belonged to the class 2 type V-A CRISPR/Cas system (Zetsche et al., 2015). Cas13a was a member of the type VI locus system and the sole determinant for RNA guidance (Myhrvold et al., 2018). These variances might indicate why preincubation only improved the Cas13a biosensing system but not the Cas12a system (Figure 5D).

### Detection Antibody and the anti-IgG–ssDNA Probe Incubation Sequence

The sequence of adding the detection antibody and probe to the reaction system was investigated. The detection antibody was pre-mixed with the anti-IgG–ssDNA probe and then added to the reaction system together. However, the signal level was lower than the standard protocol (Figure 5E). The signal decreased after modifying the protocol because the structure of the complex of the secondary antibody with the probe affects recognizing and binding of the analytes with the detection antibody. Antibodies are Y-shaped molecules consisting of two heavy chains (H chains) and two light chains (L chains) (Graves et al., 2020). An analyte binds to the antigen-binding site at the tip of the “Y” (Graves et al., 2020). In the modified protocol, the IgG–ssDNA probe recognized the detection antibody and constituted the complex “detection antibody + probe” by binding the tip of the detection antibody light chain. So, it might decrease the ability of the detection antibody to bind the analytes (Figure 5E).

### Clinical Sample Validation

**FIGURE 6:**
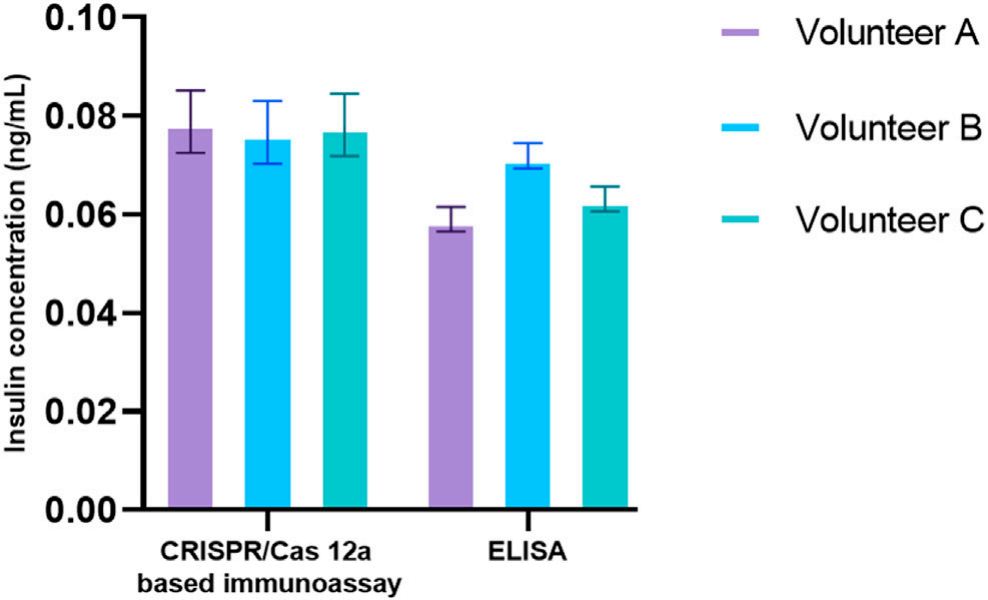
Detection of salivary insulin from volunteers both by a CRISPR/Cas12a-assisted new immunoassay (CANi). The antibody pair #1 and triggering ssDNA 1, and guiding RNA 1 were used in the assay.

Saliva had been progressively studied as a non-invasive and relatively stress-free diagnostic alternative to blood (Desai and Mathews, 2014). Elevated fasting insulin was a hallmark of insulin resistance (Hayashi et al., 2013). Salivary insulin presented a non-invasive way and a unique opportunity to monitor insulin levels before disease progression. After optimization of all reaction conditions, the performance of the CANi designed here was validated by the detection of salivary insulin. Three volunteer’s saliva samples were collected. The concentration of salivary insulin from three volunteers detected by CANi was comparable to that by insulin ELISA, although it was slightly higher than that by ELISA (Figure 6). That indicated that CANi was sensible, accurate, and reliable when detecting human salivary insulin in the clinical samples. (Figure 6).

## Conclusion

In this study, we designed a CANi system, which provides a general detection system for a wide range of analytes. When using insulin as a model molecule, specifically factors affecting the assay performance were investigated. It shows that the higher concentration BSA had a better block effect. It was also found that the detection sensitivity for the analytes was different between two matched antibody pairs, suggesting correct antibody pairs are essential to the performance of CANi. The sequence of guiding RNA had a significant impact on this assay’s efficiency among these factors studied herein. The sensitivity of the immunoassay decreased to half after changing the sequence of guiding RNA. Factors such as preincubation of Cas12a protein and guiding RNA, the extended incubation time after adding Cas12a working solution, and adding the mixture of the detection antibody and anti-IgG-ssDNA probe did not affect the effectiveness of CANi. Finally, the proposed CANi proved to be highly sensible and accurate for detecting salivary insulin with the sensitivity of 10 fg/ml and a linear range from 10 fg/ml to 1 ng/ml, which will be beneficial for early diagnosis and prevention of insulin resistance–related diseases. Additionally, this CANi system is general and suitable for all analytes that immunoassays can detect. This study guides the future design of CRISPR/Cas-based immunoassay for sensitive detection of low-abundant analytes.

## Materials and Methods

### Reagents and Instruments

Goat anti-mouse IgG (GTX77316, GeneTex), goat anti-rabbit IgG (GTX77061, GeneTex), streptavidin (85,878, Sigma Aldrich), streptavidin conjugation kit (ab102921, Abcam), Amicon® Ultra filters low-binding PES filter with 30 k molecular separation pores (UFC503096, Millipore), agarose powder (4,718, Sigma Aldrich), TAE buffer (Tris/Acetic Acid/EDTA) (1,610,743, Bio-Rad), SYBG Gold dye (41,003, Thermo Fisher Scientific), low–molecular weight DNA ladder (B7025, New England Biolabs Inc.), PowerPac™ basic power supply (1,645,050, Bio-Rad), Mini-Sub Cell GT Cell (1,704,406, Bio-Rad), Gel Doc™ EZ System (1708270EDU, Bio-Rad), SpectraMax iD5 Multi-Mode Microplate Reader (Molecular Devices), recombinant human insulin (I2643, Sigma Aldrich), recombinant human IFN-γ (285-IF, R&D Systems), recombinant human IP10 (ab9810, Abcam), recombinant IL-6 (206-IL, R&D Systems), recombinant TNF-α (210-TA, R&D Systems), recombinant IGF-1 (291-G1, R&D Systems), recombinant human proinsulin C-peptide (NBP235211, Novus Biologicals), rabbit polyclonal anti-human insulin (ab53591, Abcam), rabbit polyclonal anti-human human insulin antibody (NBP1-87485, Novus Biologicals), rabbit polyclonal anti-human insulin (biotin) (ab53592, Abcam), mouse monoclonal anti-human insulin (ab6995, Abcam), and mouse monoclonal anti-human human insulin antibody (NBP100-73008, Novus Biologicals).

### Formation of the anti-IgG–ssDNA Probe

The anti-IgG–ssDNA probe was synthesized according to the protocol of the streptavidin conjugation kit. First, 1 µL of the kit modifier was gently mixed with 10 µL (1 mg/ml) of the anti-IgG antibody (66.7 pmol). Then 10 µL (1 mg/ml) streptavidin (189.4 pmol) was added into the solution and gently mixed at room temperature (RT) for 3 h. After that, 1 µL of the quencher reagent was gently mixed into the solution for 30 min at RT. Then the biotinylated triggering ssDNA (Supplementary Table S1) was added into the mixture with a ratio of 10 µL of 10 µM (100 pmol) biotinylated triggering ssDNA together with 1 µg of the previously prepared streptavidin-conjugated antibody (6.7 pmol) for 3 h at RT. Finally, the solution was centrifugation-filtered using a low-binding PES filter with 30 k molecular separation pores by centrifuging at 12,000 rpm for 5 min and repeat for 3 times, to remove the unattached triggering ssDNA. The anti-IgG-ssDNA probe was stored at 4°C for further use.

### Electrophoretic Mobility Shift Assay (EMSA)

The agarose powder was completely dissolved in the 1 × TAE (Tris/acetic acid/EDTA) buffer (pH 8.3) to make 2% agarose gel. The gel solution was cooled down to ∼60°C before adding 0.01% (v/v) of 100,00× SYBG Gold dye. Then 12 µL IgG–ssDNA probe and a low–molecular weight DNA ladder were then loaded to the wells of the gel. Electrophoresis was run at 70 V for 1 h. Finally, the gel was visualized with a Gel Doc™ EZ system for image acquisition.

### Preparation and Activation of the CRISPR/Cas12a Reaction Mixture

First, 10 µL of 10 µM Cas12a protein (100 pmol) was gently mixed with 5 µL of 20 µM guiding RNA (100 pmol, Supplementary Table S1) in 5 ml 1 × NEB 2.1 buffer. Then 10 µL 100 µM (1 nmol) pre-synthesized ssDNA linked fluorescent reporter (the sequence was listed in the Supplementary Table S1) was added and mixed to form the final reaction mixture.

### Fabricate the Analyte Capture Surface on the 96-Well Plate

First, 50 µL of the capture antibodies (the concentrations of each antibody used in the experiments are listed in the Supplementary Table S3) were immobilized on a 96-well high-binding polystyrene plate at 4°C overnight. Next, the surfaces were blocked using BSA (50 mg/ml) at RT for 1 h and then washed by 0.01 M phosphate-buffered saline (PBS) three times. Finally, the sensing interface was stored at 4°C for further detections.

### Detection of the Analytes of Interest by Using an Immunoassay Based on CRISPR/Cas12a

Analytes (50 µL) were added to the previously prepared ELISA plate wells and incubated for 1 h at RT. Information of all the analytes used in the experiments is reported in the Supplementary Table S2. Then the wells were washed with 200 µL PBS-T (0.2% Tween 20 in PBS) three times. Next, 50 µL of detection antibodies (Supplementary Table S3) were incubated for another 1 h at RT. After that, the wells were washed with PBS-T three times. Afterward, the anti-IgG-ssDNA probe (50 μl, 4 μg/ml) was incubated at RT for 1 h. After washing the wells with PBS-T for three times, the prepared CRISPR/Cas12a (4 μg/ml) reaction mixture was added to the wells (50 µL per well) and incubated for 1 h at RT. The SpectraMax iD5 Multi-Mode Microplate Reader was used to detect the fluorescence signal with Ex = 570 nm and Em = 615 nm.

## Preparation of Saliva Samples

Under the ethic permission number HC190300, saliva samples were collected. The saliva samples were obtained by spontaneous salivation in sterilized tubes and stored at −20°C. The samples were centrifuged at 1,500 g for 10 min at RT. The supernatant was transferred to a clean tube, and 50 μl supernatant was pipetted into assay tubes for the insulin assay.

## Data Availability

The original contributions presented in the study are included in the article/Supplementary Material; further inquiries can be directed to the corresponding author.
