# Supplementary material for: Study on Factors Affecting the Performance of a CRISPR/Cas-Assisted New Immunoassay: Detection of Salivary Insulin as an Example
Source: Front Bioeng Biotechnol. 2021 Nov 11;9:752514. doi: 10.3389/fbioe.2021.752514 (PMC8632245; doi:10.3389/fbioe.2021.752514)
Supplement: Supplementary file 1 [file DataSheet1.PDF]

Study on factors affecting the performance of a CRISPR/Cas assisted new immunoassay: detection of salivary insulin as an example

Xiaoting Lin,[1] Gonglei Wang,[2] Long Ma,[3] Guozhen Liu[1,2*]

[1]Graduate School of Biomedical Engineering, University of New South Wales, Sydney NSW 2052, Australia

[2]School of Life and Health Sciences, The Chinese University of Hong Kong, Shenzhen 518172, P.R. China

[3]State Key Laboratory of Food Nutrition and Safety, Key Laboratory of Industrial Microbiology, Ministry of Education, Tianjin Key Laboratory of Industry Microbiology, National and Local United Engineering Lab of Metabolic Control Fermentation Technology, China International Science and Technology Cooperation Base of Food Nutrition/Safety and Medicinal Chemistry, College of Biotechnology, Tianjin University of Science & Technology, Tianjin, 300457, China.

Supplementary information

Table S1. The sequences of ssDNA and ssRNA using in the system

Name	Oligo type	Sequence 5'-3'	Length (nt)	Modification
**Triggering ssDNA 1**	ssDNA	ACA CAA CCA CCC AAC ACA ACC AAC CCC	27	3'-biotin
**Triggering ssDNA 2**	ssDNA	GAA GAC ACC CTA CCA ACC CCC CCC ACC ACC	30	3'-biotin
**Guiding RNA (gRNA) 1**	ssRNA	UAA UUU CUA CUA AGU GUA GAU GGG GUU GGU UGU GUU GGG UGG	42	N/A
**Guiding RNA (gRNA) 2**	ssRNA	UAA UUU CUA CUA AGU GUA GAU GGG GGG GGU UGG UAG GGU GUC	42	N/A
**ssDNA reporter**	ssDNA	TTATT	5	5'-Texa Red; 3'-BHQ2

Table S2. The analytes used in the system

Name	Cat. No.	Company
**Recombinant human insulin**	I2643	Sigma Aldrich
**Recombinant human IFN-γ**	285-IF	R&D Systems
**Recombinant human IP10**	Ab9810	Abcam
**Recombinant IL-6**	206-IL	R&D Systems
**Recombinant TNF-α**	210-TA	R&D Systems
**Recombinant IGF-1**	291-G1	R&D Systems
**Recombinant human proinsulin C-Peptide**	NBP235211	Novus Biologicals

Table S3. Antibodies used in the system

	Name	Cat. No.	Company	Concentration (μg/mL)
**Capture antibody**	Rabbit polyclonal anti-human insulin (Pair #1)	Ab53591	Abcam	10
	Rabbit polyclonal anti-human human insulin antibody (Pair #2)	NBP1-87485	Novus Biologicals	10

	Rabbit polyclonal anti-human insulin (biotin)	Ab53592	Abcam	10
**Detection antibody**	Mouse monoclonal anti-human insulin (Pair #1)	Ab6995	Abcam	10
**IgG Antibodies**	Mouse monoclonal anti-human human insulin antibody (Pair #2)	NBP100-73008	Novus Biologicals	10
**Antibody for probe fabrication**	Goat anti-mouse IgG	GTX77316	GeneTex	1000
	Goat anti-rabbit IgG	GTX77061	GeneTex	1000

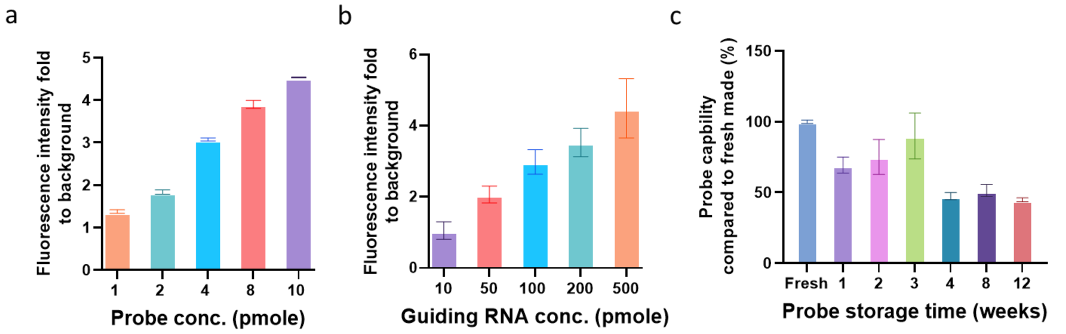

Fig. 1S Optimisation of the concentrations of anti-IgG-ssDNA probe (a), guiding RNA (b) and assessment of anti-IgG-ssDNA probe storage time (c).

The concentrations of the anti-IgG-ssDNA probe and guiding RNA had been optimised. With increasing the concentration, the signal levels in CANi promoted. Applied 4 pmole of the probe and 100 pmole guiding RNA in the CANi, the signal levels reached more than 3 times as the blank control indicated 4 pmole and 100 pmole could be used in the future experiment. Additionally, anti-IgG-ssDNA probe storage time was assessed. Until 12 weeks after the probe was conjugated, the signal level could still keep 50 % as the freshly made probe.

a

Name	Oligo type	Sequence
Guide RNA 1	ssRNA	5’-UAA UUU CUA CUA AGU GUA GAU GGG GUU GGU UGU GUU GGG UGG-3’
Trigger DNA 1	ssDNA	5’-ACA CAA CCA CCC AAC ACA ACC AAC CCC-3’

b

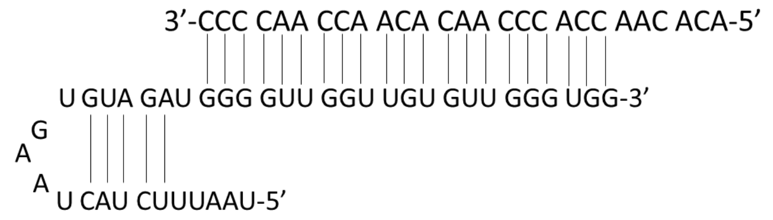

Fig. S2 The sequences (a) and secondary structures of guide RNA 1 and Trigger DNA 1 (b).

a

Name	Oligo type	Sequence
Guide RNA 2	ssRNA	5’-UAA UUU CUA CUA AGU GUA GAU GGG GGG GGU UGG UAG GGU GUC -3’
Trigger DNA 2	ssDNA	5’-GAA GAC ACC CTA CCA ACC CCC CCC ACC ACC-3’

b

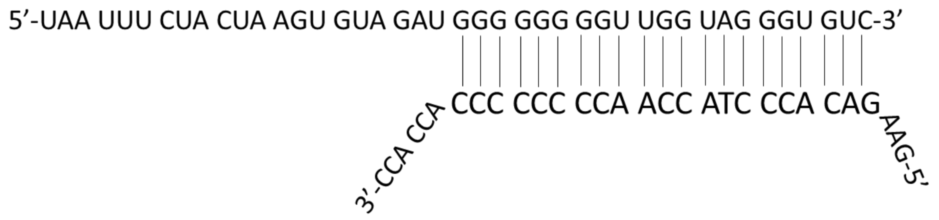

Fig. S3 The sequences (a) and secondary structures of guide RNA 2 and Trigger DNA 2 (b).
